# Poster Session I - A142 EXAMINING FACTORS CONTRIBUTING TO GENDER PAY DIFFERENCES AMONG ONTARIO PHYSICIANS IN SUBSPECIALTIES OF INTERNAL MEDICINE, INCLUDING GASTROENTEROLOGY - AN UPDATE

**DOI:** 10.1093/jcag/gwaf042.142

**Published:** 2026-02-13

**Authors:** M Bushra, F Balderrama, L Sibley, N Bollegala

**Affiliations:** Gastroenterology, University of Toronto, Toronto, ON, Canada; Ontario Medical Association, Toronto, ON, Canada; Ontario Medical Association, Toronto, ON, Canada; Women’s College Hospital, Toronto, ON, Canada

## Abstract

**Background:**

Prior research has shown that gender pay inequity exists among physicians, with little improvement in the last few years. A study examining the physician gender pay gap in Ontario showed that women physicians earned 13.5% less than men daily, even after correcting for factors such as practice characteristics, specialty and rurality^1^

**Aims:**

To identify factors contributing to gender-based differential billing among physicians in Ontario, with a specific focus on providers in procedural sub-specialties of internal medicine including gastroenterology

**Methods:**

We performed a cross-sectional study using data from the Ontario Health Insurance Plan (OHIP) in the 2021 and 2022 fiscal years. OHIP billings were used to estimate differences in gross payments between men and women physicians in procedural subspecialties of internal medicine, including gastroenterology, cardiology and respirology. Factors examined included procedural days, part-time work, after hours work and practice setting. Physicians who submitted claims to OHIP were included. Chi-square test was used to calculate differences between categorical variables, and t-test for continuous variables.

**Results:**

A total of 3600 physicians were included in the study sample (1212 [33.7%] women and 2388 [66.3%] men. . A higher percentage of female sub-specialists practiced in an urban setting compared to males (74% vs 70%). Male physicians in procedural subspecialties earned an average of $391,734.76 more than female physicians (p < 0.001) with male gastroenterologists earning an average of $154,698.86 more (p < 0.001). Among specialties, the largest pay gap was noted in cardiology ($435,000). On average, men had 4.3 more years since medical school graduation compared to women (p < 0.01), with the difference being 5.0 years among male and female gastroenterologists (p < 0.01). The total number of procedural days was higher for men (49 vs 25; p < 0.001). Male physicians in procedural subspecialties had higher percentage of days with after-hours billings (8.5 vs 5.4; p< 0.001) and weekend billings (4.2 vs 3.5; p < 0.01) compared to female physicians, and were less likely to work part-time (343 men [14%] vs 334 women [28%]; p < 0.001 Male gastroenterologists also had a higher proportion of after-hours billing (12% vs 8%; p < 0.001) compared to female gastroenterologists.

**Conclusions:**

Our study noted significant differences in gross payments between men and women physicians in procedural specialties of internal medicine including gastroenterology, which may be explained by tenure, total days worked, after-hours work and number of procedural days.

**Funding Agencies:**

None

